# Emergence of the Terrestrial Ciliate *Colpoda cucullus* from a Resting Cyst: Rupture of the Cyst Wall by Active Expansion of an Excystment Vacuole

**DOI:** 10.1264/jsme2.ME12145

**Published:** 2012-12-27

**Authors:** Ryoji Funadani, Yasutaka Suetomo, Tatsuomi Matsuoka

**Affiliations:** 1 Department of Biological Science, Faculty of Science, Kochi University, Kochi 780–8520, Japan; 2 Iwakuni City Microlife Museum, Iwakuni 740–1488, Japan

**Keywords:** *Colpoda*, excystment, excystment vacuole, emergence

## Abstract

The first sign of excysting *Colpoda cucullus* cells is the initiation of the pulsation of a contractile vacuole, which is then replaced by a non-pulsating vacuole (excystment vacuole) that continues to expand and finally ruptures the outermost cyst wall (ectocyst) due to inner pressure. A ciliate surrounded by flexible membranes (endocyst) thus emerges. The osmolarity of the excysting cells is estimated to be 140 mOsm L^−1^ from the relationship between the frequency of contractile vacuole pulsation and the external sucrose concentration. Both the expansion of the excystment vacuole and the emergence of ciliates occurred even when the cysts were immersed in hypertonic medium. In hypotonic medium containing sodium azide (NaN_3_, a cytochrome c oxidase inhibitor), the contractile vacuole of vegetative cells stopped pulsating and gradually expanded, causing cells to burst. When *C. cucullus* was induced to encyst in a hypotonic medium containing NaN_3_, the expansion of the excystment vacuoles was inhibited. These results suggest that the active uptake of water may be responsible for the expansion of the excystment vacuole required for the ectocyst to rupture.

Resting cyst formation (encystment) and sporulation are common survival strategies in terrestrial unicellular organisms. In the terrestrial ciliate *Colpoda cucullus*, the formation of resting cysts that are resistant to drying and to high or low temperatures (9, 13) can be rapidly induced by vegetative cells suspended in a Ca^2+^-containing food-free medium at high cell density (10), while excystment is induced by the addition of plant extract (1, 7, 14) including the infusion of wheat leaves (15), or sodium copper chlorophyllin (15). Two different types of emergence of motile cells from cysts in the final stage of the excystment process have been outlined by Corliss and Esser (5), and the ciliate species and their emergence modes were summarized by Müller, based on the literature (11, and references therein). One mode is emergence through an emergence pore with a removable plug, and the other takes place through the rupture of an outer cyst wall by inner pressure due to the expansion of a large vacuole known as an ‘excystment vacuole’ (11). In many ciliates, such as *Didinium nasutum* (8), *Histriculus cavicola* (12), *Meseres corlissi* (11), *Nassula ornate* (3), *Oxytricha fallax* (6), and *Tillina magna* (2), the emergence of motile cells from a cyst is performed by the latter mode. Specifically, a motile cell enclosed in a flexible thinner membrane, which is presumed to be an ‘endocyst’ (2), exits through the rupture in the outermost cyst wall. The cells struggle to escape and finally the surrounding membrane is broken (2, 11). It has been suggested that the excystment vacuole may originate from a contractile vacuole that stops water expulsion (3). Could the outermost cyst wall (ectocyst) then rupture by the elevation of inner pressure resulting from passive water inflow? If so, neither the expansion of the excystment vacuole nor the emergence of motile cells would occur under isotonic or hypertonic conditions. In the present study, we examined the excystment process of *C. cucullus*, whose emergence mode is the second of those described above, and suggest that the expansion of the excystment vacuole is caused by active uptake of water.

## Materials and Methods

*Colpoda cucullus* (strain Nag-1) was cultured in a 0.05% (w/v) infusion of dried wheat leaves inoculated with a non-pathogenic strain of bacteria (*Klebsiella pneumoniae*). The vegetative cells were induced to encyst by keeping them in encystment-inducing medium containing 1 mM Tris-HCl (pH 7.2) and 0.1 mM CaCl_2_ at high cell density (20,000–30,000 cells mL^−1^). In order to induce excystment, wet resting cysts 3 days old or older that had adhered to the bottom of vessels were soaked in 1 mM Tris-HCl (pH 7.2) containing 0.1 mM sodium copper chlorophyllin (chlorophyllin-Cu) (Fig. 2) or in a 0.2% infusion of dried wheat leaves (Figs. 3–6).

**Fig. 1:**
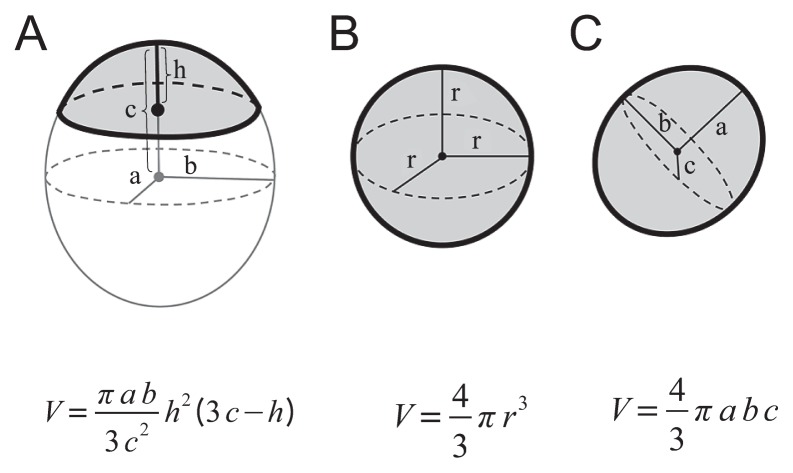
Calculation of the volumes of the excystment vacuole (A), contractile vacuole (B) and cell (C). Each volume (shaded portion) was calculated using the formulas given at the bottom of each figure.

Excystment-induced cysts were transferred to a microscope slide, and the excysting process was observed with an Olympus BX-51 differential interference contrast microscope equipped with a CCD video camera (Victor JVC, KY-F550; Olympus), or with an Olympus BX-50 microscope equipped with a camera (Coolpix; Nikon). For convenience, we regarded the shapes of the excystment vacuole, contractile vacuole and resting cyst as an ellipsoidal cap, a sphere and an ellipsoid, respectively; therefore, the volumes of these bodies were calculated using the formula shown in Fig. 1 (http://keisan.casio.jp/).

## Results and Discussion

**Fig. 2:**
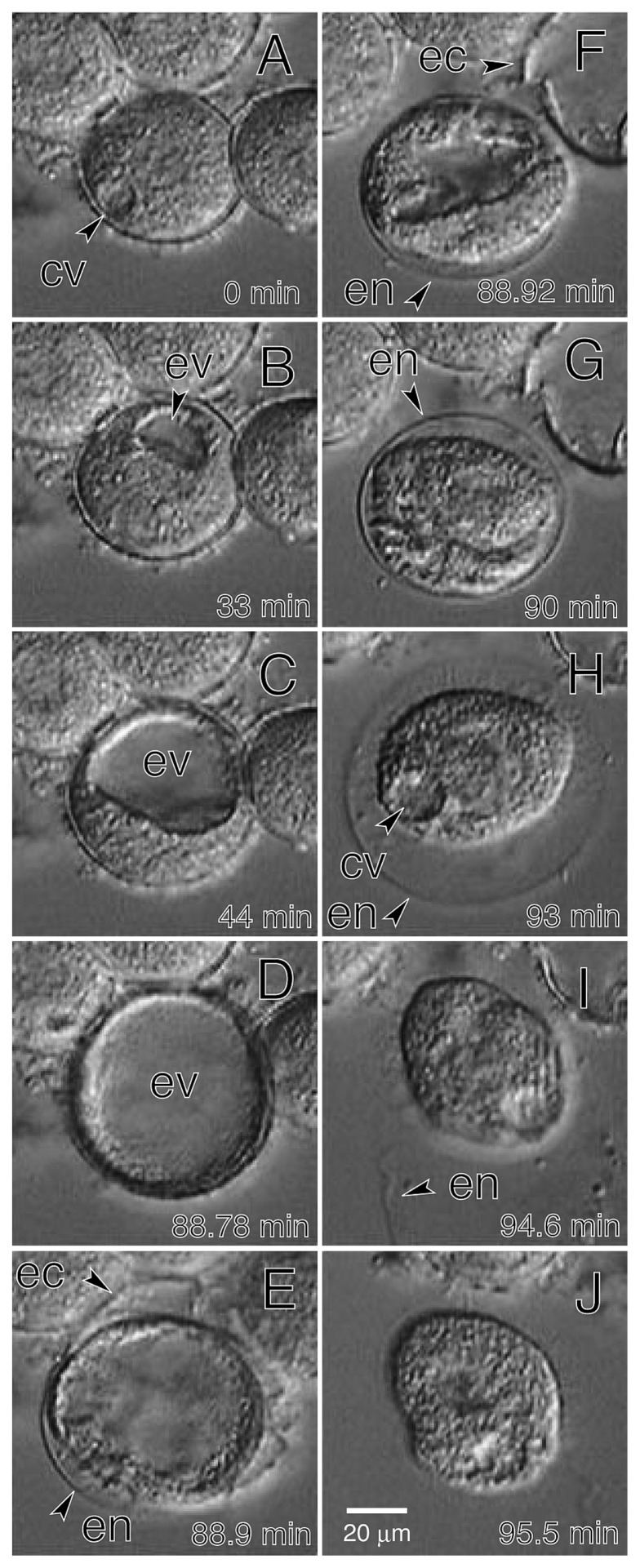
Emergence of a *C. cucullus* motile cell from a resting cyst. The cell was induced to encyst in medium containing 1 mM Tris-HCl (pH 7.2) and 0.1 mM chlorophyllin-Cu. Video-recorded images were captured at 0 (A), 33 (B), 44 (C), 88.78 (D), 88.9 (E), 88.92 (F), 90 (G), 93 (H), 94.6 (I) and 95.5 min (J) after the contractile vacuole began to pulsate. cv, contractile vacuole; ev, excysting vacuole; en, endocyst; ec, ectocyst.

Fig. 2 shows the emergence of motile cells from a *C. cucullus* resting cyst induced to excyst by immersion in 0.1-mM chlorophyllin-Cu solution. The pulsation of a contractile vacuole, which was preceded by cyclosis for several minutes, began at 30 min in the earliest case after the onset of excystment induction. The pulsation of a contractile vacuole (Fig. 2A, ‘cv’) stopped approximately 30 min after it began to pulsate, and a non-pulsating vacuole (‘excystment vacuole;’ Fig. 2B, ‘ev’) appeared instead of the contractile vacuole. The encystment vacuole gradually expanded (Fig. 2B–E) until the outermost layer (ectocyst; Fig. 2E, ‘ec’) of the cyst wall ruptured (Fig. 2E). As soon as the cell, surrounded by a flexible inner membrane that may be an endocyst (Fig. 2E, ‘en’), emerged from the ruptured cyst, the excystment vacuole quickly shrank (Fig. 2E–G), and the pulsating contractile vacuole reappeared (Fig. 2H, ‘cv’). As described in *M. corlissi* (11), the membrane surrounding a motile cell is extended by the movement of the ciliate until it finally bursts open (Fig. 2G–I) and the cell swims out (Fig. 2J).

**Fig. 3:**
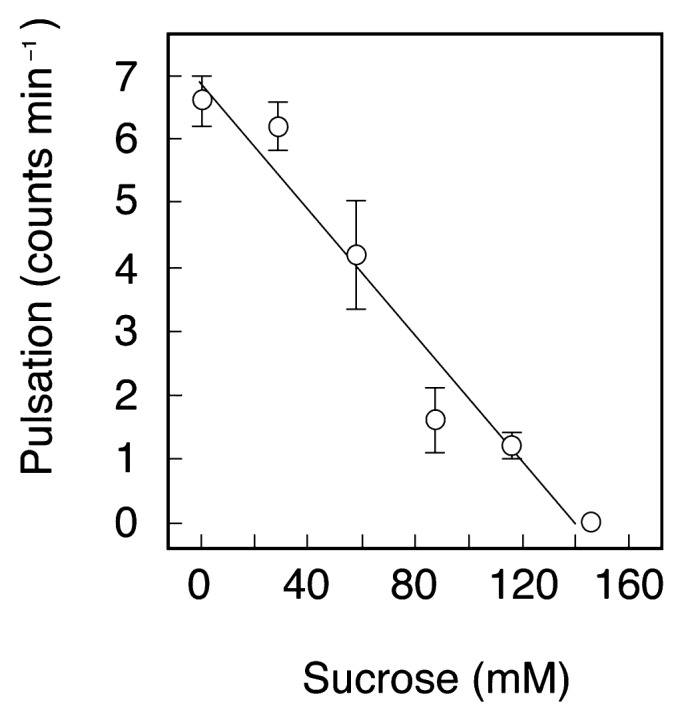
Pulsation frequency of the contractile vacuoles of excysting *C. cucullus* cells measured immediately after the initiation of pulsation. The points and attached bars correspond to the means and SE, respectively, of measurements obtained using 5 different specimens.

In order to elucidate whether the expansion of the excystment vacuole is caused by the passive diffusion of water into the cell interior, the growth of the excystment vacuole was observed under hypertonic conditions. Prior to observation, the intracellular osmolarity of excysting cells was estimated from the relationship between the extracellular sucrose concentration (containing 0.2% wheat leaf infusion) and the pulsation frequency of the contractile vacuole (Fig. 3). The pulsation frequency of the contractile vacuole decreased with increasing osmolarity of the surrounding medium, and stopped finally at a sucrose concentration of 140 mM (Fig. 3). Based on this result, the osmolarity of the excysting cells was estimated to be 140 mOsm L^−1^, although in fact, the intracellular osmolarity is slightly higher, because the test solution contained 0.2% wheat leaf infusion whose osmolarity was 4.2 mOsm L^−1^ (determined by the freezing point depression method).

**Fig. 4:**
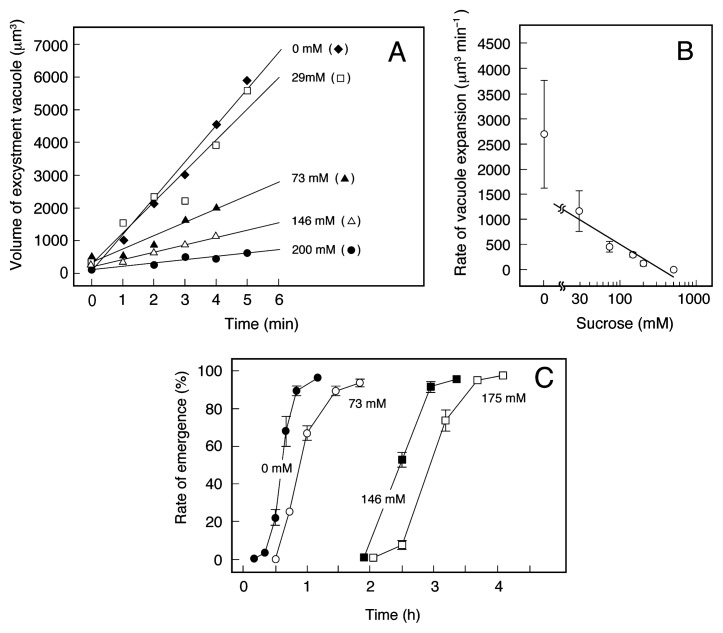
Effect of osmolarity on the expansion of the excystment vacuole and emergence rate (%) in excystment-induced cysts of *C. cucullus*. (A) Typical time course of the increase in the volume of the excystment vacuole of the excysting cells, which were induced to excyst in media containing various concentrations of sucrose and a 0.2% infusion of wheat leaves. (B) Relationship between external sucrose concentration and the expansion rate (μm^3^ min^−1^) of the excystment vacuole obtained from Fig. 4A. Points and attached bars correspond to the means and SE of 5 identical measurements, respectively. (C) Effect of external sucrose concentration on the emergence rate (%) of motile cells. In this assay, 100–300 cysts were randomly chosen, and the rate of vacant cysts (those from which the cells had already emerged) was expressed as a percentage of the total number of cells. Points and attached bars correspond to the means and SE, respectively, of 5 identical measurements.

If only passive water diffusion into the cell interior is responsible for the expansion of the excysting vacuole, causing the rupture of the ectocyst, its expansion and the emergence of ciliates are expected not to occur under isotonic or hypertonic conditions. Fig. 4A shows the typical time course of the elevation of the volume of the excystment vacuole when cysts were induced to excyst in media containing various concentrations of sucrose. Fig. 4B shows the relationship between external osmolarity and the expansion rate of the excysting vacuole obtained from Fig. 4A. The expansion rate of the excystment vacuole decreased as external osmolarity increased (Fig. 4B). The time required for the emergence of ciliates increased with increasing external osmolarity (Fig. 4C). As shown in Fig. 4A–C, the excystment vacuole expanded and motile cells emerged (Fig. 4C), even when the cysts were placed in hypertonic medium (more than 140 mOsm L^−1^). These results suggest that active uptake of water is involved in the expansion of the excystment vacuole.

**Fig. 5:**
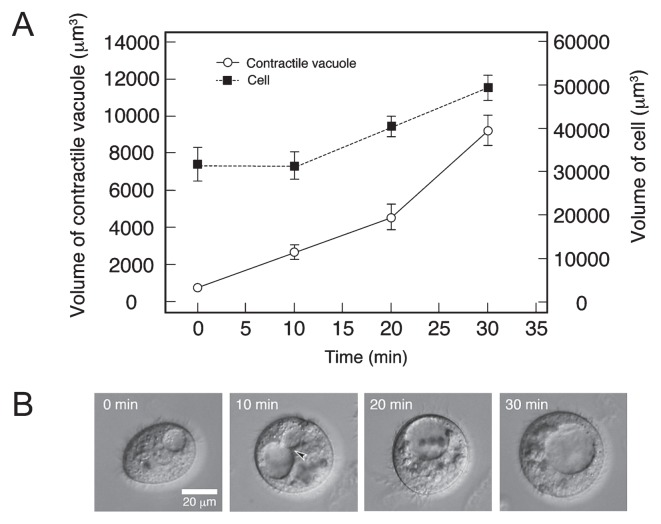
Effects of 1 mM NaN_3_ on contractile vacuole activity in *C. cucullus* vegetative cells. (A) Time course of the increase in the volumes of whole cells (closed squares) and contractile vacuoles (open circles) after the cells were placed in a 1-mM NaN_3_ solution. (B) Photomicrographs of NaN_3_-treated vegetative cells. Photomicrographs were taken at 0, 10, 20, and 30 min after the cell was transferred into a 1-mM NaN_3_ solution. Arrow indicates water accumulation where a small vacuole was observed to fuse with a non-pulsating contractile vacuole.

If the active uptake of water is involved in the expansion of the excystment vacuole, it is expected to be suppressed by the inhibition of adenosine triphosphate (ATP) synthesis. In vegetative cells, a contractile vacuole was stopped from pulsating and gradually expanded by the addition of 1 mM sodium azide (NaN_3_), an inhibitor of cytochrome c oxidase (4) (Fig. 5A, B). In this case, water seemed to continue to be collected in the vacuole via fusion with a small vacuole (Fig. 5B, arrowhead). The cell volume reached 1.5-fold its original volume, which is the cell burst limit, within 30 min after the contractile vacuole stopped pulsating (Fig. 5A, B). These results indicate that the water-gathering system of the contractile vacuole may function even in the presence of 1 mM NaN_3_, and that passive inflow of water generates sufficient inner pressure to burst the vegetative cell.

**Fig. 6:**
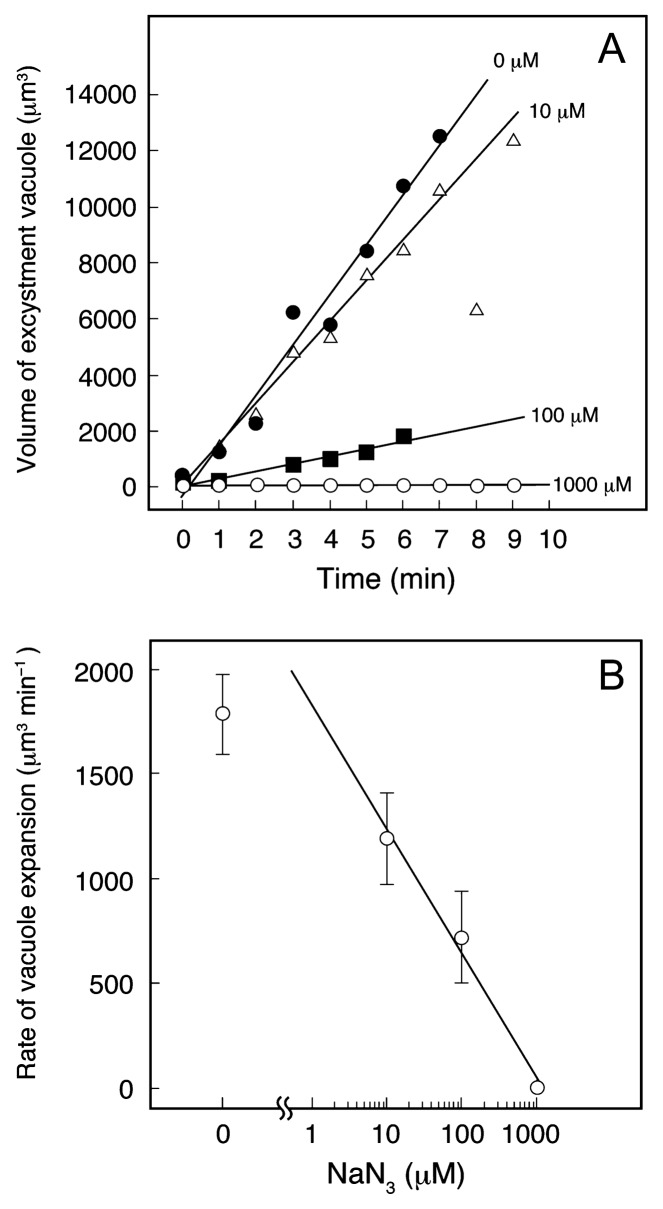
Effects of NaN_3_ on the expansion of the excystment vacuole in excystment-induced *C. cucullus*. (A) Typical time course of the increase in vacuole volume of excysting cells, which were induced to excyst in media containing a 0.2% infusion of wheat leaves and various concentrations of NaN_3_. Measurement began immediately after the appearance of the excystment vacuole (at about 30 min after excystment induction). In 1-mM NaN_3_ solution, the excystment vacuole did not appear at least within 3 h. (B) The relationship between the rate of vacuole expansion (μm^3^ min^−1^, obtained from Fig. 6A) and NaN_3_ concentrations. Points and attached bars correspond to the means and SE, respectively, of 5 identical measurements.

Fig. 6 shows the effect of NaN_3_ on the expansion of the excystment vacuole of cells that have been induced to excyst in media containing a 0.2% infusion of wheat leaves and various concentrations of NaN_3_. In the presence of 1 mM NaN_3_, the expansion of the excystment vacuole was completely suppressed. In this case, it was also difficult to increase cell volume (data not shown), indicating that in excysting cells, neither an increase in cell volume nor the expansion of an excystment vacuole can be caused solely by the passive inflow of water. If water passively flows into the cell interior, the power of the water diffusion may soon be balanced by the turgor pressure of the rigid cyst wall. These results suggest that active uptake of water may be involved in the expansion of an excystment vacuole.

The manner of expansion of a non-pulsating contractile vacuole of NaN_3_-treated vegetative cells mimicked the process of expansion of an excystment vacuole. During excystment, the excystment and contractile vacuoles never coexist. This implies that the excystment vacuole may be derived from the contractile vacuole, as has been suggested in other ciliates such as *N. ornate* (3).
